# Detecting Brain Structure-Specific Methylation Signatures and Rules for Alzheimer’s Disease

**DOI:** 10.3389/fnins.2022.895181

**Published:** 2022-05-02

**Authors:** ZhanDong Li, Wei Guo, Tao Zeng, Jie Yin, KaiYan Feng, Tao Huang, Yu-Dong Cai

**Affiliations:** ^1^College of Food Engineering, Jilin Engineering Normal University, Changchun, China; ^2^Key Laboratory of Stem Cell Biology, Shanghai Institutes for Biological Sciences, Chinese Academy of Sciences, Shanghai Jiao Tong University School of Medicine, Shanghai, China; ^3^Bio-Med Big Data Center, CAS Key Laboratory of Computational Biology, Shanghai Institute of Nutrition and Health, Chinese Academy of Sciences, University of Chinese Academy of Sciences, Shanghai, China; ^4^Cancer Institute, Second Affiliated Hospital, Zhejiang University School of Medicine, Hangzhou, China; ^5^Department of Human Genetics, Institute of Genetics, Zhejiang University School of Medicine, Zhejiang University, Hangzhou, China; ^6^Department of Computer Science, Guangdong AIB Polytechnic College, Guangzhou, China; ^7^CAS Key Laboratory of Tissue Microenvironment and Tumor, Shanghai Institute of Nutrition and Health, Chinese Academy of Sciences, University of Chinese Academy of Sciences, Shanghai, China; ^8^School of Life Sciences, Shanghai University, Shanghai, China

**Keywords:** Alzheimer’s disease, brain structure, DNA methylation, machine learning, quantitative rules

## Abstract

Alzheimer’s disease (AD) is a progressive disease that leads to irreversible behavioral changes, erratic emotions, and loss of motor skills. These conditions make people with AD hard or almost impossible to take care of. Multiple internal and external pathological factors may affect or even trigger the initiation and progression of AD. DNA methylation is one of the most effective regulatory roles during AD pathogenesis, and pathological methylation alterations may be potentially different in the various brain structures of people with AD. Although multiple loci associated with AD initiation and progression have been identified, the spatial distribution patterns of AD-associated DNA methylation in the brain have not been clarified. According to the systematic methylation profiles on different structural brain regions, we applied multiple machine learning algorithms to investigate such profiles. First, the profile on each brain region was analyzed by the Boruta feature filtering method. Some important methylation features were extracted and further analyzed by the max-relevance and min-redundancy method, resulting in a feature list. Then, the incremental feature selection method, incorporating some classification algorithms, adopted such list to identify candidate AD-associated loci at methylation with structural specificity, establish a group of quantitative rules for revealing the effects of DNA methylation in various brain regions (i.e., four brain structures) on AD pathogenesis. Furthermore, some efficient classifiers based on essential methylation sites were proposed to identify AD samples. Results revealed that methylation alterations in different brain structures have different contributions to AD pathogenesis. This study further illustrates the complex pathological mechanisms of AD.

## Introduction

Alzheimer’s disease (AD) is a progressive disease that initiates the pathogenesis of dementia (Rogers et al., 1996; Karch and Goate, 2015). At the onset, AD symptoms are quite mild, something as seemingly innocuous as mild and gradual memory loss. However, AD can quickly progress to negatively affect not only cognitive ability but also motor skills, thereby reducing the quality of life of patients with this disease, especially the elderly (Reisberg et al., 1987; Weller and Budson, 2018; Atri, 2019). Clinically and by definition, the most common symptom of early-stage AD is a reduction in memory capacity (Weller and Budson, 2018; Atri, 2019). As AD progresses, it may also lead to irreversible behavioral changes, erratic emotions, and loss of motor skills. These conditions make people with AD difficult or almost impossible to take care of.

In 2014, over 5 million people suffered from AD in the United States (Murphy et al., 2015; Taylor et al., 2017). In general, AD symptoms manifest after 65 years of age. Moreover, the incidence of suffering from this disease increases with age, indicating that the sooner the first AD symptoms appear, the more severe the disease would be. Another study estimated that over 200,000 people in the United States are already suffering from advanced-stage AD (Matthews et al., 2019), suggesting that the people who develop this disease are younger than 65 years old and the symptoms are more severe than they should be. These trends make AD one of the most serious public health concerns.

Multiple internal and external pathological factors may affect or even trigger the initiation and progression of AD. DNA methylation is one of the most effective regulatory roles during AD pathogenesis (Mastroeni et al., 2010; De Jager et al., 2014; Qazi et al., 2018). A systematic review summarized the specific roles of epigenetic changes, including methylation alterations, during AD pathogenesis (Mastroeni et al., 2010). Methylation participates in the advancement of multiple progressive diseases, such as cancer and AD (Mastroeni et al., 2010). Multiple demethylation alterations further affect AD pathogenesis *via* regulating downstream proteins, such as *DNMT1*, *MBD2*, and *p66-alpha*, especially in specific brain structures, i.e., the entorhinal cortex (ERC; Mastroeni et al., 2010). This review indicated that methylation contributes to AD pathogenesis in specific brain structures.

Other studies also explored potentially pathological methylation alterations in the different brain structures of people with AD. Methylation status seems to consistently decrease in the hippocampus (HIPPO) during AD progression (Chouliaras et al., 2013). Other researchers focused on the status of methylation in the middle temporal gyrus (Coppieters et al., 2014). Unlike in the HIPPO, DNA modifications as implied by levels of 5-mc and 5-hmc continuously increase in the middle temporal gyrus of patients with AD. Another team identified specific methylation statuses at potentially effective genes, such as *ANK1, BIN1*, and *RHBDF2*, which have a specific methylation status during AD initiation (De Jager et al., 2014). Their results implied that some unique genomic loci rather than the entire genome may also be examined to effectively monitor and predict AD progression. The methylation of various candidate genes at specific brain structures may be strongly associated with AD progression and can be potentially used in clinically predicting AD pathogenesis and progression.

Although multiple loci associated with AD initiation and progression have been identified, the spatial distribution patterns of DNA methylation in the brain of people with AD have not been clarified. A team systematically analyzed the status of methylation in different brain structural regions from more than 60 samples (Semick et al., 2019). The said study provides us four comprehensive datasets for exploring alterations in methylation status in various brain regions at loci that are potentially associated with AD. As the powerful machine learning algorithms can deeply analyze hidden and complicated relationships in a big dataset (Dai et al., 2016; Kong et al., 2020; Yang et al., 2020), we adopted them to investigate above-mentioned comprehensive datasets to identify a group of candidate loci potentially associated with AD that regulate methylation and show structural specificity. In our previous studies, we analyzed tissue-specific methylation that is associated with the pathogenesis of complex diseases, such as cancer (Pan et al., 2019) and viral infections (Chen et al., 2019). Herein, we extend our understanding of methylation in specific tissues by profiling the methylation of brain diseases, which in this case is AD. The above-mentioned four datasets were related to four major brain structures: dorsolateral prefrontal cortex (DLPFC), HIPPO, ERC, and cerebellum (CRB). Previous studies confirmed that these structures are correlated with brain functions associated with AD: decision making and working memory (DLPFC; Heekeren et al., 2006), spatial memory and navigation (HIPPO; Broadbent et al., 2004), neuron information processing and recording (ERC; Keene et al., 2016), and learning (CRB; Raymond et al., 1996). This study focused on these structures because of their functional correlations with AD pathogenesis. On each dataset, we applied several multiple machine learning algorithms to investigate it. The Boruta feature filtering (Kursa and Rudnicki, 2010) and max-relevance and min-redundancy (mRMR; Peng et al., 2005) methods were performed one by one. Some essential methylation features were extracted and ranked in a feature list. This list was fed into the incremental feature selection (IFS; Liu and Setiono, 1998) method that integrated three classification algorithms: random forest (RF; Breiman, 2001), support vector machine (SVM; Cortes and Vapnik, 1995), partial decision tree (PART) (Frank and Witten, 1998), to identify candidate AD-associated methylation features with structural specificity, set up a group of quantitative rules, which can reveal the effects of DNA methylation in various brain regions (i.e., four brain structures) on AD pathogenesis. In addition, on each brain region, some efficient classifiers were built to identify AD samples. Our results revealed that methylation alterations in different brain structures have different contributions to AD pathogenesis. This study further illustrates the complex pathological mechanisms of AD.

## Materials and Methods

### Data

The DNA methylation profiles of AD and control samples from postmortem brain donors’ four brain regions: CRB, DLPFC, ERC, and HIPPO, were downloaded from Gene Expression Omnibus under accession number of GSE125895 (Semick et al., 2019). In CRB dataset, there were 24 AD and 43 control samples. In DLPFC dataset, there were 21 AD and 47 control samples. In ERC dataset, there were 20 AD and 49 control samples. In HIPPO dataset, there were 17 AD and 48 control samples. The DNA methylation levels were measured with Illumina HumanMethylation450 BeadChip and represented with normalized beta values processed with the R/Bioconductor package minfi^1^. The processed data of 420,852 methylation probes in 269 samples were used for further analysis.

### Boruta Feature Filtering

As mentioned in section “Data,” all samples in four datasets were represented by a large number of methylation features. Evidently, not all of these features give contributions for identification of AD samples. It is necessary to exclude irrelevant features first.

To quick complete this task, we employed the Boruta feature filtering (Kursa and Rudnicki, 2010). It is a powerful feature selection method based on RF. The Boruta method involves several steps. First, shuffled data from copies of the original data are created. Second, the RF is trained on the original and shuffled data to obtain feature importance. Third, the *Z* score for each feature is computed on the basis of this feature’s importance score. Fourth, a feature is tagged as important if and only if its *Z* score is greater than that of shadow features. Finally, these steps are repeated for all features. The features tagged as important are outputted as the outcome of this method, which are deemed to be relevant features.

In this study, we adopted the Boruta program obtained from https://github.com/scikit-learn-contrib/boruta_py, which was performed on each dataset with its default parameters. Selected features would be analyzed by the following mRMR method.

### Max-Relevance and Min-Redundancy Feature Selection

For the remaining methylation features, we further used mRMR method (Peng et al., 2005) to analyze them. This method calculates the mutual information (MI) between features and class labels for evaluating feature relevance and MI between features to assess the redundancy between features. Its original purpose was to find out a feature subset that had maximum relevance to class labels and minimum redundancies between features in this set. However, such problem is NP-hard. In view of this, it adopts a heuristic way. In this way, investigated features are sorted in a feature list, where important features are assigned high ranks. In the beginning, this list is empty. The method repeatedly selects one features from the remaining features such that it has maximum relevance to class labels and minimum redundancies to features already in the list. Such procedure stops until all features are in the list. This list is called mRMR feature list.

The present study used the mRMR program retrieved from http://penglab.janelia.org/proj/mRMR/. It was applied on each dataset, in which samples were represented by features selected by Boruta. Accordingly, one mRMR feature list was obtained on each dataset.

### Incremental Feature Selection

Although mRMR method deeply analyzed the importance of each methylation feature, it was still a problem which features can be selected. Thus, we employed the IFS method (Liu and Setiono, 1998) to determine the optimal number of features according to the integrating supervised classification algorithm. In brief, a series of feature subsets are extracted from the mRMR feature list yielded by the mRMR method at a step interval of 1. For instance, the first feature subset has the top ranked feature, and the second feature subset has the top 2 ranked features, and so on. For each feature subset, a classifier is trained based on the training data induced from this feature subset and the given classification algorithm. All classifiers are evaluated by 10-fold cross-validation (Kohavi, 1995). When all classifiers have been assessed, the best one can be discovered. This classifier is called the optimal classifier and the features used in such classifier are termed as the optimal features.

### Classification Algorithm

To execute the IFS method, one classification algorithm is necessary. In this study, we used the following three classification algorithms: RF (Breiman, 2001), SVM (Cortes and Vapnik, 1995), and PART (Frank and Witten, 1998).

#### Random Forest

Random forest is widely applied in analyzing biological and biomedical data. Several previous studies indicate the satisfactory performance of RF (Pan et al., 2010; Zhao et al., 2018; Jia et al., 2020; Chen et al., 2021, 2022; Ding et al., 2022; Li Z. et al., 2022; Wu and Chen, 2022; Zhou et al., 2022). RF is a meta-classifier because it consists of numerous decision trees. Each decision tree in RF is learned from a bootstrap training data induced from a randomly selected feature subset. For an input sample, each decision tree provides its prediction. RF integrates these predictions by majority voting. To quickly implement RF, the tool “RandomForest” in Weka (Frank et al., 2004) was adopted. For convenience, such tool was executed using default parameters.

#### Support Vector Machine

Support vector machine is a classic and powerful classification algorithm. At present, it is always an important candidate for constructing efficient classifiers (Chen et al., 2017; Zhou et al., 2020a,b; Liu et al., 2021; Zhu et al., 2021; Li X. et al., 2022). In mathematical terms, the main task of SVM is to transform non-linear data from the original low-dimensional data space to a new high-dimensional data space, where the new data would be linear as guaranteed by a certain kernel trick. SVM then determines support vectors on the data margin between samples from two classes, which can represent a hyperplane in high-dimensional data space to predict the class type of new samples. Likewise, we also used the tool “SMO” in Weka (Frank et al., 2004) to implement SVM. Such SVM is optimized by the sequence minimization optimization algorithm (Platt, 1998).

#### PART Rule Learning

Based on one of above two algorithms, investigators can build efficient classifiers. However, they are absolute black-box algorithms. Their classification principles are difficult to be understood. Few clues can be extracted from them to uncover differences between AD and control samples at the methylation level. In view of this, a rule learning algorithm, PART (Frank and Witten, 1998) was employed. Different from other rule learning algorithms, PART can learn rules at a time. Hence, PART is a simpler and more efficient approach without global optimization. In general, PART can produce many partial decision trees and combine the rules from these decision trees *via* the separate-and-conquer technique. Similar to RF and SVM, we also used one tool in Weka to implement PART. The tool was named as “PART.”

### Synthetic Minority Oversampling Technique

In each dataset, control samples were about twice as many as AD samples. It is better to balance samples in these two classes. To this end, the widely used method, synthetic minority oversampling technique (SMOTE; Chawla et al., 2002) was adopted, which is an over-sampling method. It iteratively produces new samples for the minor class until the minor class has equal number of samples in major class. In detail, it first randomly selects a sample, say *x*, in the minor class. Then, a number of samples in this class are picked up such that these samples have smallest distance to *x*. The randomly selected sample, say *y*, from above samples and *x* are used to generate the new sample *z*, which is defined as the linear combination of *x* and *y*. The tool “SMOTE” in Weka was applied in this study. The SMOTE was only used in evaluation of performance of classifiers. The samples generated by it did not participate in evaluating the importance of methylation features.

### Performance Evaluation

Matthew correlation coefficients (MCC) is a widely used measurement for binary classification (Matthews, 1975; Zhao et al., 2019; Liang et al., 2020; Zhang et al., 2021b; Yang and Chen, 2022), which was adopted herein to assess the performance of the different classifiers within a 10-fold cross-validation (Kohavi, 1995; Zhao et al., 2019; Zhang et al., 2021a; Tang and Chen, 2022). The MCC can be computed as follows:


(1) $$M\,C\,C=\frac{T\,P\times T\,N-F\,P\times F\,N}{\sqrt{\left(T\,P+F\,P\right)\,\left(T\,P+F\,N\right)\,\left(T\,N+F\,P\right)\,\left(T\,N+F\,N\right)}},$$

where *TP*, *TN*, *FP*, and *FN* indicate the sample number of true-positive, true-negative, false-positive, and false-negative predictions, respectively. MCC values range from -1 to +1 and achieves +1 when the classification model has the best performance.

In addition, we also calculated other five measurements: sensitivity (SN), specificity (SP), prediction accuracy (ACC), precision and F1-measure, to fully display the performance of different classifiers. They can be computed by


(2) $$S\,N=\frac{T\,P}{T\,P+F\,N}$$

(3) $$S\,P=\frac{T\,N}{T\,N+F\,P}$$

(4) $$A\,C\,C=\frac{T\,P+T\,N}{T\,P+T\,N+F\,P+F\,N}$$

(5) $$P\,r\,e\,c\,i\,s\,i\,o\,n=\frac{T\,P}{T\,P+F\,P}$$

(6) $$F\,1-m\,e\,a\,s\,u\,r\,e=\frac{2\times T\,P}{2\times T\,P+F\,N+F\,P}$$

## Results

**FIGURE 1:**
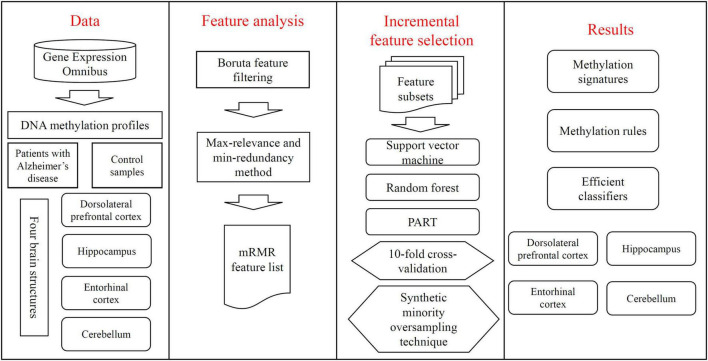
Entire procedures of the investigation on DNA methylation profile of patients with Alzheimer’s disease and control samples. The profile is retrieved from Gene Expression Omnibus. Four datasets are constructed, corresponding to four brain structures. Each dataset is deeply analyzed by Boruta feature filtering and max-relevance and min-redundancy methods one by one, resulting in a feature list. This list is used in the incremental feature selection, containing some classification algorithms, to identify methylation signatures, rules and construct efficient classifiers.

In this study, the DNA methylation dataset on four brain structures of AD and control samples was deeply analyzed. The entire procedures are illustrated in Figure 1. This section gave the detailed results produced by such procedures.

### Results of Boruta and Max-Relevance and Min-Redundancy Methods

For each brain structure, one methylation dataset was collected. As a large number of methylation features were involved in each dataset, the Boruta method was first applied on each dataset to exclude irrelevant features. Important methylation features remained for each dataset, which are provided in Supplementary Table 1. The number of remaining features on each dataset is listed in Table 1. It can be observed that less than 200 features were kept on each dataset, greatly reducing the scope of investigated methylation features. These features were deemed to be essential for distinguishing AD patients from control samples on corresponding brain structure.

**TABLE 1:** Number of methylation features selected by Boruta method on each dataset.

Dataset on brain structure	Number of methylation features
CRB dataset	124
ERC dataset	158
DLPFC dataset	149
HIPPO dataset	132

The features selected by Boruta method were further analyzed by the mRMR method according to Figure 1. One mRMR feature list was produced on each dataset, which is also provided in Supplementary Table 1. Such list would be investigated in the following analysis.

### Incremental Feature Selection Results on Four Brain Regions

The mRMR method produced an mRMR feature list on each dataset, corresponding to one brain structure. Then, the IFS method was applied on such list, which integrated three classification algorithms.

**FIGURE 2:**
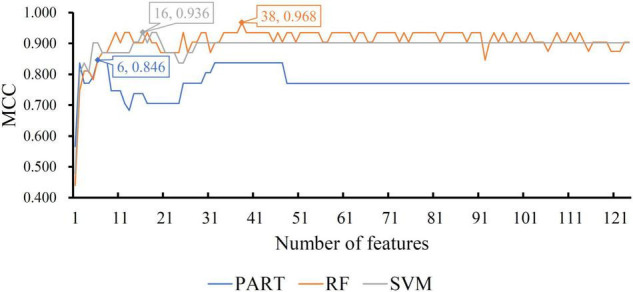
IFS curves with different classification algorithms on different number of methylation features for CRB brain structure. The RF, SVM, and PART yield the highest MCC of 0.968, 0.936, and 0.846, respectively. They are obtained by using top 38, 16, and 6, respectively, features in the list.

**FIGURE 3:**
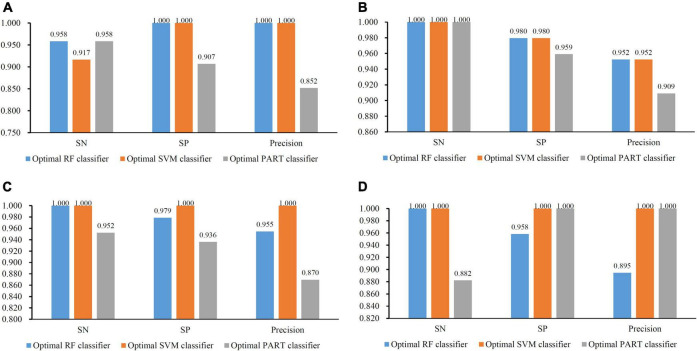
Bar chart to show three measurements of the optimal classifiers on four brain structures. **(A)** CRB brain structure; **(B)** ERC brain structure; **(C)** DLPFC brain structure; and **(D)** HIPPO brain structure. The optimal PART classifier generally provided the lowest performance.

For the CRB dataset, the mRMR feature list contained 124 methylation features. Accordingly, 124 feature subsets were generated in the IFS method by using step one. On each subset, a classifier was built with one of the three classification algorithms, which was further assessed by 10-fold cross-validation. The predicted results were counted as measurements listed in section “Performance Evaluation,” which are provided in Supplementary Table 2. To clearly display the performance of the classifiers based on one classification algorithm and different feature subsets, one IFS curve was plotted for each classification algorithm by setting MCC as *Y*-axis and number of used features as *X*-axis, as shown in Figure 2. It can be observed that the RF, SVM, and PART produced the highest MCC values of 0.968, 0.936, and 0.846, respectively. These values were obtained by using top 38, 16, and 6, respectively, features in the mRMR feature list. These features were the optimal ones for corresponding classification algorithm. Accordingly, the optimal RF/SVM/PART classifier can be built using corresponding optimal features. The ACC and F1-measure of these classifiers are listed in Table 2, whereas the other three measurements: SN, SP, and precision, are illustrated in Figure 3A. Evidently, the optimal RF classifier was best, followed by the optimal SVM classifier and optimal PART classifier.

**TABLE 2:** Performance of the optimal classifiers based on different classification algorithms on four brain structures.

Dataset of brain structure	Classification algorithm	Number of features	ACC	MCC	F1-measure
CRB dataset	RF	38	0.985	0.968	0.979
	SVM	16	0.970	0.936	0.957
	PART	6	0.925	0.846	0.902
ERC dataset	RF	1	0.986	0.966	0.976
	SVM	4	0.986	0.966	0.976
	PART	2	0.971	0.934	0.952
DLPFC dataset	RF	6	0.985	0.967	0.977
	SVM	20	1.000	1.000	1.000
	PART	2	0.941	0.868	0.909
HIPPO dataset	RF	15	0.969	0.926	0.944
	SVM	18	1.000	1.000	1.000
	PART	34	0.969	0.920	0.938

**FIGURE 4:**
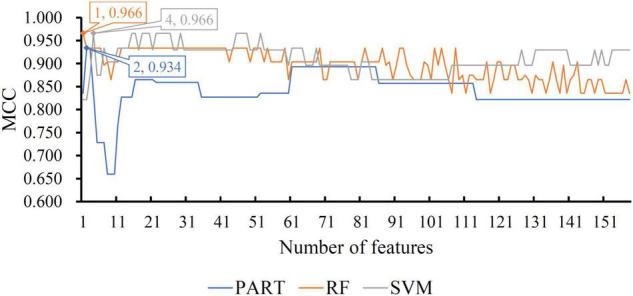
IFS curves with different classification algorithms on different number of methylation features for ERC brain structure. The RF, SVM, and PART yield the highest MCC of 0.966, 0.966, and 0.934, respectively. They are obtained by using top 1, 4, and 2, respectively, features in the list.

**FIGURE 5:**
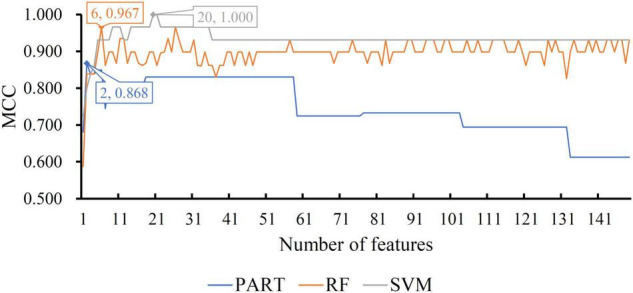
IFS curves with different classification algorithms on different number of methylation features for DLPFC brain structure. The RF, SVM, and PART yield the highest MCC of 0.967, 1.000, and 0.868, respectively. They are obtained by using top 6, 20, and 2, respectively, features in the list.

**FIGURE 6:**
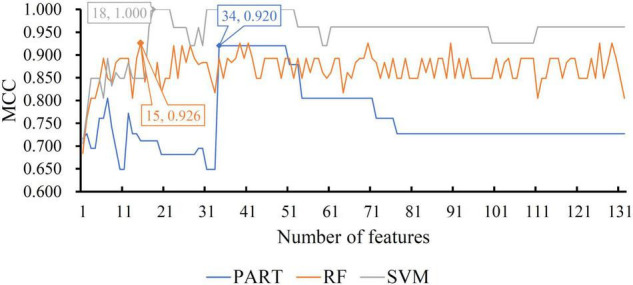
IFS curves with different classification algorithms on different number of methylation features for HIPPO brain structure. The RF, SVM, and PART yield the highest MCC of 0.926, 1.000, and 0.920, respectively. They are obtained by using top 15, 18, and 34, respectively, features in the list.

For the other three datasets (ERC, DLPFC, and HIPPO datasets), the same IFS procedures were conducted. Detailed IFS results are provided in Supplementary Tables 3–5. Three IFS curves, corresponding to three classification algorithms, were plotted, as shown in Figures 4–6. From Figure 4 for ERC dataset, we can see that RF/SVM/PART yielded the highest MCC of 0.966/0.966/0.934, which was based on top 1/4/2 features in the list. Accordingly, we can build three optimal classifiers with corresponding features. Other measurements of these classifiers are provided in Table 2 and Figure 3B. Clearly, the optimal RF and SVM classifiers gave equal performance, whereas they were all superior to the optimal PART classifier. For the IFS results on DLPFC dataset, it can be observed from Figure 5 that three classification algorithms generated the highest MCC values of 0.967, 1.000, and 0.868, respectively. Top 6, 20, and 2, respectively, features in the list were adopted to produce these MCC values, which comprised the optimal features for the corresponding classification algorithm. With these optimal features, three optimal classifiers were built. Their detailed performance is listed in Table 2 and Figure 3C. The optimal SVM classifier gave the best performance, followed by the optimal RF and PART classifiers. As for the last dataset (HIPPO dataset), the highest MCC value for RF/SVM/PART was 0.926/1.000/0.920, which can be observed from Figure 6. This MCC was obtained by using top 15/18/34 features in the list. They were optimal features of corresponding classification algorithm. The optimal RF/SVM/PART classifier was constructed with its optimal features. Table 2 and Figure 3D summarizes the detailed performance of these three optimal classifiers. Evidently, the optimal SVM classifier was best, whereas other two optimal classifiers were almost at the same level.

Based on the above IFS results, we can select a best classifier on each dataset, which can be an efficient tool for identification of AD samples based on a certain brain structure.

### Classification Rules on Four Brain Regions

On each brain structure, the optimal PART classifier always provided the lowest performance. However, it can provide more clues than other two optimal classifiers to uncover different methylation patterns within a certain brain structure of AD patients and control samples. By employing the optimal features used in the optimal PART classifier, PART learned rules based on all AD and control samples. These rules are listed in Table 3. Two rules were obtained for CRB brain structure. For other three brain structures, four, two and two, respectively, rules were accessed. These rules clearly displayed the different patterns on methylation levels between AD and control samples, which were helpful to improve our understanding on AD.

**TABLE 3:** Classification rules generated by PART for four brain structures.

Brain structure	Rule index	Condition	Result
CRB	Rule-1	(cg15926585 > 0.2202) AND (cg08105590 > 0.1467) AND (cg07094785 > 0.7870)	Control
	Rule-2	Others	AD
ERC	Rule-1	cg05810363 ≤ 0.8365	Control
	Rule-2	cg05810363 > 0.8793	AD
	Rule-3	cg08462501 > 0.8234	AD
	Rule-4	Others	Control
DLPFC	Rule-1	(cg14622549 ≤ 0.8740) AND (cg21097354 ≤ 0.2427)	Control
	Rule-2	Others	AD
HIPPO	Rule-1	(cg13076843 > 0.8321) AND (cg06639320 > 0.3468)	AD
	Rule-2	Others	Control

## Discussion

In this study, several computational methods were adopted to analyze the DNA methylation profile of AD and control samples. As pathological methylation alterations may occur in the different brain structures, the methylation profile on four brain structures was investigated. Some key methylations and rules were obtained, as listed in section “Results.” Here, the analyses on some methylations and rules were conducted.

### Methylation Signatures and Rules From the Cerebellum

Cerebellum mainly contributes to learning and the establishment of theories and computational models. Given that a reduction in learning capacity is a typical symptom of AD (Sultana et al., 2006), identifying the methylation alterations during AD pathogenesis in the CRB is necessary. In our prediction list, the top methylation locus was **cg07094785**, which targets *HNRNPUL2*. Although only a few studies directly examined the function of this gene, it reportedly participates in AD pathogenesis, for example, by regulating the accumulation of tau protein in brain (Vanderweyde et al., 2016). Given that CRB tissues are highly specific, this gene was found to effectively function during nerve system degeneration due to age in multiple animal models, including mice and rats (Jasien et al., 2014; Kalinin et al., 2017). The other top-ranked locus was **cg08105590**, which targets *FAM38A*. This locus can also be associated with AD pathogenesis in the CRB at the methylation level. This gene might be correlated with AD by regulating Piezo1 channels (Velasco-Estevez et al., 2018), which has been shown to have a tight correlation with AD initiation and progression. Given that such correlations were validated using cerebellar cells (Velasco-Estevez et al., 2018), we can reasonably speculate that they can be observed in the CRB *in vivo*.

The first parameter for quantitative rules is **cg15926585**, which targets the intermediate region of *COMT* and *TXNRD2*. These two genes are both highly negatively correlated with the abnormal behavioral symptoms of AD (Borroni et al., 2006; Jin et al., 2017). Methylation of this region may inhibit the expressions of these genes and further trigger AD initiation and progression. Two other parameters that target *HNRNPUL2* and *FAM38A* are functionally correlated with AD. Hypermethylation of these genes has also been demonstrated to participate in the development of diseases associated with nerve disorders (Pathak et al., 2019; Gürkan et al., 2020) but not with AD. Therefore, these quantitative rules in the CRB were explained according to publications above, demonstrating the effectiveness of our analysis.

### Methylation Signatures and Rules From the Entorhinal Cortex

Entorhinal cortex is a region that controls memory, navigation, and time perception (Goyal et al., 2018; Montchal et al., 2019). ERC is reportedly correlated with AD initiation and progression (Marzi et al., 2018; Grubman et al., 2019; Petrache et al., 2019). In our predicted feature list, the first locus is **cg05810363**, which targets *RHBDF2*. This gene is hypermethylated in HIPPO cells during AD pathogenesis (De Jager et al., 2014). Similar methylation patterns were recently identified in the ERC *via* interacting with *H3K27AC* (Marzi et al., 2017; Marzi et al., 2018). The methylation status of another locus, namely, **cg08462501**, which interacts with *TBC1D1*, is predicted to be downregulated during AD pathogenesis. Given that the ERC is correlated with a specific biomarker called TDP-43, this region could be hypomethylated during AD pathogenesis (Sun et al., 2017). Therefore, *TBC1D1* may also present methylation status in the ERC similar to that in HIPPO.

Three quantitative rules, including the two features discussed in this section, i.e., the hypermethylated gene *RHBDF2* and the hypomethylated gene *TBC1D1*, may contribute to AD pathogenesis. This contention was supported by previous reports.

### Methylation Signatures and Rules From Dorsolateral Prefrontal Cortex

Dorsolateral prefrontal cortex mainly controls the decision-making and working memory functions of the human brain. Given that people with AD show a decrease in the ability to render judgment (Heekeren et al., 2006) and a decline in working memory at late stages (Mars and Grol, 2007), we can reasonably regard this region as a potential target to identify methylation effects associated with AD pathogenesis. A locus predicted by our computational analysis is **cg14622549**, which targets the gene body region of *EP400*. As early as 2013, *EP400* has already been regarded as a potential biomarker for detecting AD at the transcriptomics level (Desire et al., 2013; Trifonova et al., 2022). Methylation of *EP400* is reportedly functionally correlated with autism, another disease of the nervous system that affects DLPFC. This supposition suggests the potential pathological effects of *EP400* and supports our contention that methylation affects specific brain structures. The next locus is **cg21097354**, which targets the intermediate region of *TTYH3* and *AMZ1*. *AMZ1* has been shown to be a potential regulator for nerve system, especially in the elderly (Swingler et al., 2009; Panossian et al., 2018). Given that AD is a typical disease of the nervous system that is associated with aging, we can reasonably predict the potential relationships between DLPFC and AD. On the basis of a monozygotic twin model, a study suggested that the demethylation of *AMZ1* in DLPFC is associated with dyskinesia, another potential symptom of AD (Soerensen et al., 2019). This report further confirmed the pathogenesis and downstream pathogenic regions of this methylation alteration.

The two parameters in the only classification rule were the two aforementioned loci. Their demethylation would aid in the identification of patients with AD according to this rule.

### Methylation Signatures and Rules From Hippocampus

Numerous methylation loci can help in the identification of specific methylation alterations associated with AD in HIPPO, a region that regulates memory and navigation. The first locus is **cg08056778**, which targets the 3’UTR region of *TBC1D1* and may further affect the expression of this gene in HIPPO. *TBC1D1* is correlated with cerebral palsy (Mohandas et al., 2018). Moreover, *TBC1D1* may be precisely regulated by effective microRNAs, thereby contributing to the development and aging of the central nervous system (Sephton et al., 2012). An *in vivo* study based on the model organism *Caenorhabditis elegans* confirmed that *TBC1D1* in HIPPO may be functionally correlated with aging-related diseases (Escoubas-GüNey, 2018). Although this study did not cover AD, it nevertheless confirmed the tissue specificity of this gene. Thus, we can reasonably argue that pathological methylation alterations in HIPPO may affect AD pathogenesis, thereby validating our prediction. Similarly, *SDK1*, *ANK1*, and *CHRNB4*, which are targeted by **cg04680535**, **cg05066959**, and **cg17179314**, respectively, also have either a certain gene expression level regulated by a specific methylation status (De Jager et al., 2014; Kanno et al., 2014; Yan et al., 2019) or an epigenomic change during AD pathogenesis (Li et al., 2010; De Jager et al., 2014).

Two parameters associated with the extracted quantitative rules were predicted to aid in the identification of patients with AD. The first parameter is *RHBDF2* (**cg13076843**), which has already been confirmed to be associated with AD (De Jager et al., 2014). The other parameter, that is, **cg06639320**, was also screened to be hypermethylated in patients with AD. The targeting of *FHL2* by this locus is reportedly correlated with AD progression (Bennett et al., 2017; Altuna et al., 2019).

## Conclusion

This study is the first to identify a group of brain structure-specific AD signatures and quantitative rules at the methylation level. According to previous studies, the identified signatures and the parameters associated with this rule are correlated with AD pathogenesis. Therefore, the present study successfully screened a group of effective molecules with pathological potentials. This study also established a systematic workflow for the identification of effective tissue-specific biomarkers for complex diseases.

## Data Availability Statement

Publicly available datasets were analyzed in this study. This data can be found here: https://www.ncbi.nlm.nih.gov/geo/query/acc.cgi?acc=GSE125895.

## Author Contributions

TH and Y-DC designed the study. TZ and KF performed the experiments. ZL, WG, and JY analyzed the results. ZL, WG, and TZ wrote the manuscript. All authors contributed to the research and reviewed the manuscript.

## Conflict of Interest

The authors declare that the research was conducted in the absence of any commercial or financial relationships that could be construed as a potential conflict of interest.

## Publisher’s Note

All claims expressed in this article are solely those of the authors and do not necessarily represent those of their affiliated organizations, or those of the publisher, the editors and the reviewers. Any product that may be evaluated in this article, or claim that may be made by its manufacturer, is not guaranteed or endorsed by the publisher.

## Footnotes

1
https://bioconductor.org/packages/minfi/

## Supplementary Material

Supplementary Table 1 Lists of genes ranked by mRMR for four brain structures.

Supplementary Table 2 Performance of IFS for CRB brain structure.

Supplementary Table 3 Performance of IFS for ERC brain structure.

Supplementary Table 4 Performance of IFS for DLPFC brain structure.

Supplementary Table 5 Performance of IFS for HIPPO brain structure.
